# Flt3 Regulation in the Mononuclear Phagocyte System Promotes Ocular Neovascularization

**DOI:** 10.1155/2018/2518568

**Published:** 2018-05-03

**Authors:** Yushuo Gao, Yisheng Zhong, Yanji Zhu, Anna M. Demetriades, Yujuan Cai, Jikui Shen, Qing Lu, Xi Shen, Bing Xie

**Affiliations:** ^1^Department of Ophthalmology, Ruijin Hospital, Shanghai Jiao Tong University School of Medicine, 197, Ruijin Er Road, Shanghai 200025, China; ^2^Department of Ophthalmology, NewYork-Presbyterian Hospital-Cornell, New York, NY, USA; ^3^Departments of Ophthalmology and Neuroscience, The Johns Hopkins University School of Medicine, Maumenee 719, 600 N. Wolfe Street, Baltimore, MD, USA

## Abstract

Fms-like tyrosine kinase 3 (Flt3), a tyrosine kinase receptor expressed in CD34+ hematopoietic stem/progenitor cells, is important for both normal myeloid and lymphoid differentiation. It has been implicated in mice and humans for potential multilineage differentiation. We found that mice deficient in Flt3 or mice that received an Flt3 inhibitor (AC220) showed significantly reduced areas of ischemia-induced retinal neovascularization (RNV) and laser-induced choroidal NV (CNV) (*P* < 0.05). Increased Flt3 expression at the protein level was detected in retinas of oxygen-induced retinopathy (OIR) mice at P15 and P18 during retinal NV (RNV) progression. We subsequently found that macrophages (Mphi) polarization was regulated at the site of CNV in Flt3-deficient mice. Flow cytometry analysis demonstrated that Flt3 deficiency shifted Mphi polarization towards an M2 phenotype during RNV with significant reduction in M1 cytokine expression when compared to the wild-type controls (*P* < 0.05). Based on the above findings, we concluded that Flt3 inhibition alleviated ocular NV by promoting a Mphi polarization shift towards the M2 phenotype. Therapies targeting Flt3 may provide a new approach for the treatment of ocular NV.

## 1. Introduction

Age-related macular degeneration (AMD) represents an ailment whose incidence increases with age and is a leading cause of vision problems in older adults worldwide, affecting approximately 9% of the global population [1–3]. AMD comprises early and advanced types; the latter includes the exudative (characterized by choroidal neovascularization or CNV) and nonneovascular (distinguishable by progressive RPE atrophy) forms [2]. Neovascular AMD progresses rapidly, resulting in 80%–90% of cases with severe vision loss [1]. Currently, <40% of cases show improved vision after clinical therapy [2, 4].

In the past decades, angiogenesis and neovascularization have been studied to a great extent, allowing the development of antiangiogenic products to treat malignancies as well as ocular diseases [5]. Angiogenesis plays a critical role in development, reproduction, and repair; it involves degeneration of the vascular basement membrane, which is normally continuous, and the activation of quiescent endothelial cells (ECs) [6–8]. Vascular tubes are generated and covered with fully formed vascular basement membrane with the involvement of pericytes [7, 9, 10]. Currently, there is an increasing number of patients benefiting from angiogenesis inhibitors such as vascular endothelial growth factor (VEGF), although reduced efficacy and resistance continue to challenge the way we treat the disease [11–14]. At present, several reports have revealed novel molecular mechanisms that provide unique avenues for the improvement of antiangiogenic strategies [14–16].

Fms-like tyrosine kinase 3 (Flt3), expressed in CD34+ hematopoietic stem/progenitor cells, acts as a critical receptor for both normal myeloid and lymphoid differentiation [17]. Phosphorylation of Flt3 receptor activates the intracellular signaling pathways responsible for cell proliferation [18]. Mounting evidence indicates that inflammation, involving dendritic cells (DC) or macrophages (Mphi), accounts for important in neovascularization (NV) [19–21]. Whiteley et al. demonstrated that mesenchymal stromal and circulating angiogenic cells repair the tissues by expanding CD34+ cells [22].

In this study, we used AC220 as an inhibitor of Flt3. Prior first-generation inhibitors of Flt3 were nonselective and developed against additional targets. For instance, semaxanib and sunitinib are tyrosine kinase inhibitors with multiple targets, including VEGFR, c-KIT, and Flt3. AC220, a novel second-generation bis-aryl urea Flt3 inhibitor, has been optimized to inhibit Flt3 with high potency. It is highly selective against other kinases, together with pharmacokinetic properties that afford complete and sustained inhibition of FLT3-ITD and wild-type FLT3 *in vivo*. Moreover, it has shown favorable tolerability and single-agent activity in phase I and II trials [22–24]. Hence, this study assessed the impact of Flt3 on the differentiation of mouse Mphi and ocular NV.

## 2. Methods and Materials

### 2.1. Experimental Animals

Animal experiments were performed after approval from the Institutional Animal Care and Use Committee of Shanghai Jiao Tong University School of Medicine and carried out as directed by the Association for Research in Vision and Ophthalmology (ARVO). Pathogen-free C57BL/6 (Charles River, Wilmington, MA), Flt3−/− transgenic, rhodopsin promoter/VEGF (rho/VEGF) transgenic [16, 25], and rho/rtTA-TRE/VEGF double-transgenic [16, 26] mice were used in this study.

### 2.2. Mouse Model of Laser-Induced CNV

Laser photocoagulation rupture of Bruch's membrane was used to induce CNV in C57BL/6 as described previously [27, 28]. Briefly, laser injury (75 mm spot for 0.1 s at 120 mW) was performed with an OcuLight GL diode laser (Iridex, Mountain View, CA, USA). Only burns with bubbles were assessed and used to confirm breakage of Bruch's membrane [28].

### 2.3. Flt3 Inhibitor Injections

AC220, a second-generation Flt3 inhibitor, was purchased from Selleck (Selleckchem, Houston, TX) [23, 24]. Mice received intravitreal injections (1 *μ*l) of AC220 and phosphate-buffered saline (PBS) following laser photocoagulation (day 0, D0). This was repeated at 7 days. One microliter AC220 was administered into the vitreous cavity of murine eyes using glass micropipette needles at D0 and D7. Eye analysis was performed at 14 days in order to quantify the area of CNV.

### 2.4. CNV Flat Mount

After 14 days of modeling, the anesthetized animals underwent perfusion with 1 ml FITC-dextran (Sigma-Aldrich, St. Louis, MO, USA) for the quantification of CNV area. Next, eyes were either stored for cDNA extraction or fixed in 10% buffered formalin, enucleated, and choroid flat mounted for examination. Choroidal lesions were assessed according to previously published procedures [27, 29–31], as CNV area (*μ*m^2^) ± standard error. Groups were compared by Student's *t*-test [31].

### 2.5. Immunofluorescence Staining and Quantification

Sclerochoroidal flat mounts from CNV mice prepared on D14 after perfusion by FITC-dextran were incubated with phycoerythrin- (PE-) conjugated anti-CD11c antibody (1 : 100) and PE-conjugated anti-CD206 antibody (1 : 100) for staining. After being washed, the specimens were assessed microscopically. The samples were assessed for M1 and M2 using PE-conjugated anti-CD11c and PE-conjugated anti-CD206, respectively.

### 2.6. Quantitative Real-Time Reverse Transcriptase-Polymerase Chain Reaction (qRT-PCR)

After euthanasia of mice with or without laser-induced CNV, the eyes were removed. Total RNA in ocular cup samples was obtained by TRIzol Reagent (Invitrogen, Carlsbad, CA) as directed by the manufacturer [16, 32]. After DNase (Promega, Fitchburg, WI) pretreatment, RNA (2 *μ*g per sample) was submitted to reverse transcription for cDNA synthesis using M-MLV transcriptase and Oligo(dT) Primers (Promega) following the manufacturer's protocol. Quantitative RT-PCR was carried out according to previous reports [16, 33], with iQ SYBR Green mix (Roche, Basel, Switzerland) on an ABI 7500 system; cyclophilin was used for normalization [16, 34]. Samples from two retinas were pooled for analysis by the ΔΔCT method [16]. The following primers were utilized: CD11c, sense 5′-GTGCCCATCAGTTCCTTACA-3′ and antisense 5′-GAGAAGAACTGTGGAGCTGAC-3′; CD206, sense 5′-GGAATCAAGGGCACAGAGTTA-3′ and antisense 5′-ATTGTGGAGCAGATGGAA-3; F4/80, sense 5′-CGTCAGGTACGGGATGAATATAAG-3′and antisense 5′-CTATGCCATCCACTTCCAAGAT-3′; and cyclophilin A, sense 5′-CAGACGCCACTGTCGCTTT-3′ and antisense 5′-TGTCTTTGGAACTTTGTCTGCAA-3′.

### 2.7. Oxygen-Induced Retinal Neovascularization in Mice and Immunostaining

Wild-type, Flt3+/−, and Flt3−/− transgenic C57BL/6 mice were treated with 75 ± 3% oxygen from postnatal day 7 (P7) to P12 as discussed in previous reports [16, 35]. C57BL/6 mice were divided into several groups at P12, intravitreal injections of AC220 at 1 *μ*g/*μ*l were performed in one eye, and intravitreal PBS was injected in the contralateral eye, as described in prior studies [36, 37]. After euthanasia at P17, murine eyes were removed and formalin fixation was performed [16]. Retina samples were obtained and stained with fluorescein *Griffonia simplicifolia* lectin-B4 (GSA-Lectin; Vector Laboratories Inc., Burlingame, CA, USA, 1 : 50) as previously described [16]. Analysis was performed by fluorescence microscopy (Nikon Instruments Inc., New York, NY) with Image-Pro Plus (Media Cybernetics, Silver Spring, MD, USA) [16].

### 2.8. Phospho-Flt3 Enzyme-Linked Immunosorbent Assay

Phosphorylated Flt3 levels were assessed in mouse retina specimens obtained at P13, P15, P18, and P21 by ELISA with a specific kit as previously described [38].

### 2.9. Isolation of Mouse Retina CD11b+ cells

After intravitreal injection with 1 *μ*g/*μ*l AC220 (or control vehicle), mouse retinas at P15, P18, and P21 (6–10 retinas per group) were obtained for the isolation of retinal cells as described previously, using antimouse CD11b magnetic beads (Miltenyi Biotec, Bergisch Gladbach, Germany) and a premoistened MS column (BD Biosciences) [16].

### 2.10. Flow Cytometry Analysis

CD11b+ cells were incubated with PE-conjugated antimouse CD11c, FITC-conjugated F4/80, and Alexa Fluor 647-conjugated CD206 [16]. M1 Mphi were F4/80+/CD11c+, and M2 Mphi were F4/80+/CD206+ [16, 39]. Data analysis was performed with FlowJo (Tree Star, Ashland, OR) [16].

### 2.11. Mouse Model of VEGF Overexpression

Rho/VEGF transgenic animals were injected intravitreally with 1 *μ*g/*μ*l AC220 and PBS, in respective eyes, at P12 and submitted to perfusion with fluorescein-labeled dextran under anesthesia [16]. Eye specimens were prepared and assessed by fluorescence microscopy, with Image-Pro Plus used to evaluate the number and total area of NV lesions per retina [16].

Four- to six-week-old double-hemizygous rho/rtTA-TRE/VEGF double-transgenic animals received an intravitreal injection of 1 *μ*g/*μ*l of AC220 in one eye and intravitreal PBS in the contralateral eye. The mice then received 2 mg/ml doxycycline in drinking water [26] for 5 days. Retinas were examined under a surgical microscope to determine whether there was total retinal detachment (TRD), partial retinal detachment (PRD), or no retinal detachment (no RD) by chi-square test. C57BL/6 mice, rho/rtTA-TRE/VEGF double-transgenic mice, and double-transgenic mice treated with doxycycline were euthanized; the retinas were lysed, and phosphorylated Flt3 amounts were evaluated with PathScan Phospho-Flt3 Sandwich ELISA Kit as described previously [38, 40].

### 2.12. Immunofluorescence Staining of Flt3, GSA-Lectin, and F4/80

Immunofluorescence staining for Flt3, GSA-Lectin, and F4/80 detection was applied in normal, ischemic, rho/VEGF transgenic, CNV, and rho/rtTA-TRE/VEGF transgenic retinas. OCT- (Miles Laboratories, Elkhart, IN, USA) embedded samples were used to prepare frozen sections (10 *μ*m) [16], which were fixed in acetone at −20°C for 20 minutes and blocked with 5% bovine serum albumin (BSA). After overnight incubation at 4°C with polyclonal rabbit antimouse Flt3 antibody (Cell Signaling Technology Inc., Danvers, MA, USA), the specimens were incubated with Alexa 555 antirabbit IgG (Cell Signaling Technology), GSA-Lectin (Vector Laboratories), and FITC-labeled antimouse F4/80 antibody (eBioscience). The specimens were subsequently assessed by fluorescence microscopy.

### 2.13. Statistical Analysis

Unless otherwise stated, quantitative data are mean ± SE. The SPSS software was used for statistical analyses. Student's *t*-test, paired-sample *t*-test, chi-square test, and one-way ANOVA with the Student-Newman-Keuls (SNK) method (multiple comparisons) were used. Two-tailed *P* < 0.05 indicated statistical significance.

## 3. Results

### 3.1. Association of Flt3 with Vascular Endothelial Cells (VEC)

**Figure 1:**
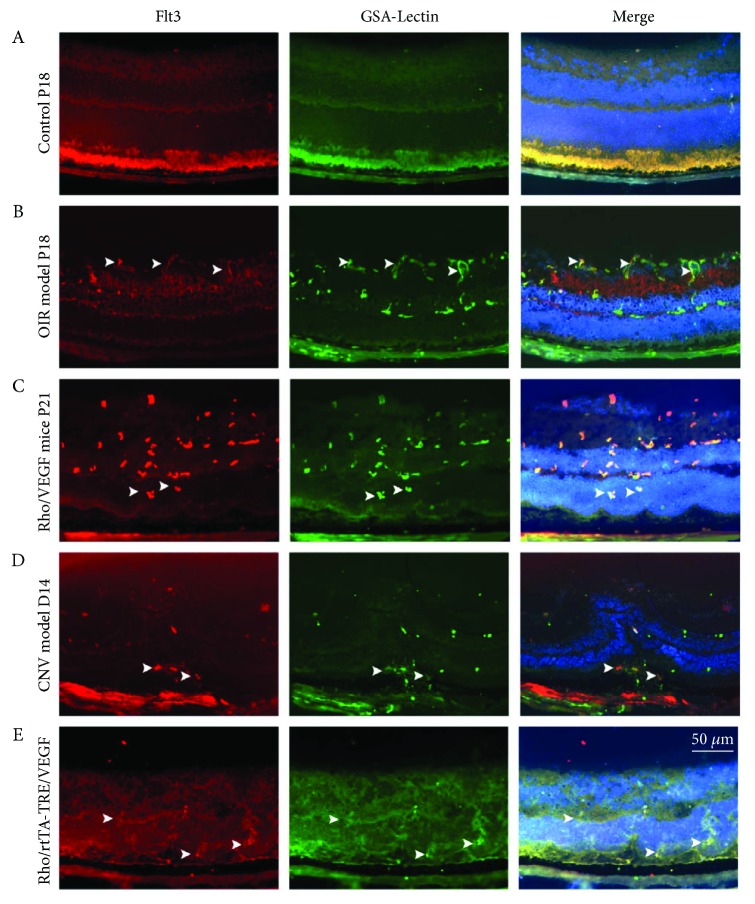
Immunofluorescence staining for Flt3 and GSA-Lectin detection in C57BL/6 control, OIR model, rho/VEGF transgenic, CNV model, and rho/rtTA-TRE/VEGF transgenic retinas. Mice were euthanized, and enucleated eyes were submitted to fixation and preparation to generate frozen sections. Immunofluorescence for Flt3 (red) and lectin (green) showed high expression of Flt3 in the OIR model, subretinal NV model, CNV model, and RD model retinas (arrowheads) with NV progression (A–E). Merged images showed colocalization of Flt3 and lectin. Scale bar: 500 *μ*m. (A) Double immunofluorescence staining of Flt3 by Cy3-conjugated secondary antibodies (red) and GSA-Lectin (green) in flat-mounted control retina at P18. (B) Double immunofluorescence staining of Flt3 (red) and GSA-Lectin (green) in flat-mounted OIR retina samples at P18. Areas of staining for Flt3 increased during the pathological progression of OIR. (C) Eye specimens from P21 transgenic mice overexpressing VEGF in photoreceptors (rho/VEGF mice) showed Flt3 staining in the inner plexiform layer and colocalization with GSA-Lectin. (D) Staining of a section from a D14 CNV model showed Flt3 staining, while GSA-Lectin staining of the same section showed vascular staining. (E) Ocular sections from 5- to 8-week-old transgenic mice (rho/rtTA-TRE/VEGF) showed increased Flt3 staining in the inner layer and a close association with vascular staining.

Retina samples from normal P18 mice showed faint staining for Flt3 and GSA-Lectin throughout the inner retina (Figure 1(A)). Retinas from P18 mice after oxygen-induced ischemic retinopathy displayed increased Flt3 staining in the inner retina with colocalization of GSA-Lectin on the retinal surface in new vessels (Figure 1(B)). Retina specimens from P21 transgenic animals overexpressing VEGF in photoreceptors (rho/VEGF mice) showed Flt3 expression in inner and intermediate layers with a close association with GSA-Lectin (Figure 1(C)). Sections from a D14 laser-induced CNV mouse showed areas of Flt3 staining closely associated with GSA-Lectin in spite of laser injury (Figure 1(D)). Eyes from 5- to 8-week-old transgenic mice (rho/rtTA-TRE/VEGF) showed areas of Flt3 staining in the inner layers, with a close association with GSA-Lectin (Figure 1(E)).

### 3.2. Levels of Flt3 in OIR Mouse Retinas

**Figure 2:**
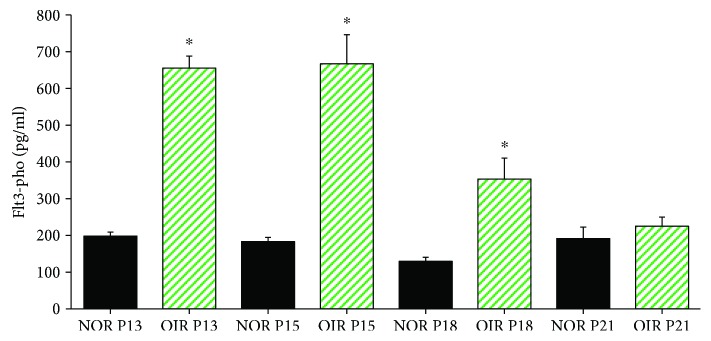
Flt3 expression examined by ELISA in retina specimens from OIR animals at P13, P15, P18, and P21. Mice subjected to OIR were sacrificed at P13, P15, P18, and P21; retinas were lysed, and phosphorylated Flt3 levels were measured as described in Methods and Materials. Quantification of phosphorylated Flt3 levels in mice retinas is shown. The asterisk “∗” indicates a significant change of phosphorylated Flt3 levels at P13, P15, and P18 in OIR mouse retina specimens compared with age-matched control samples, respectively. Bars represented mean ± SE from 3 independent experiments (*n* = 8, ^∗^*P* < 0.05).

Immunofluorescence staining and ELISA were carried out to assess whether Flt3 was involved in the progression of RNV. Phosphorylated Flt3 levels were significantly increased at P13, P15 and P18 in OIR mouse retina specimens compared with age-matched control samples (Figure 2). Immunofluorescence also revealed higher Flt3 expression in OIR mouse retina specimens at P18 (Figure 1(a)). Merged images showed colocalization of Flt3 and GSA-Lectin (Figure 1). These findings indicated that Flt3 plays an important role in RNV progression.

### 3.3. Flt3 Effects NV in Both CNV and RNV Mouse Models

**Figure 3:**
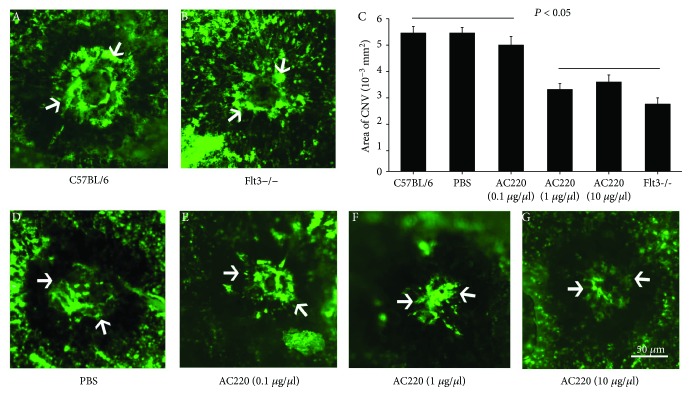
CNV at Bruch's membrane rupture sites is decreased in Flt3-deficient animals. Flt3−/− and wild-type C57BL/6 mice were submitted to laser-induced CNV. Wild-type animals were randomly grouped, with intravitreal administration of AC220 in one eye and PBS in the contralateral eye at days one and seven after laser photocoagulation in CNV mice. Choroidal flat mounts for CNV animals were obtained at day 14 (A, B, and D–G, *n* = 10). Areas of CNV were reduced at rupture sites in the Flt3−/− (B) and AC220 treatment (F) groups compared with those of C57BL/6 (A) or PBS treatment group (D). In agreement, image analysis revealed that CNV areas were markedly reduced (ANOVA with SNK) in Flt3−/− animals compared with wild-type mice. AC220-treated eyes exhibited significant inhibitory effects on CNV in comparison to PBS-treated eyes (*P* < 0.05) (C). Dose-effect experiments revealed that 1 *μ*g/*μ*l AC220 reduced CNV significantly at the lowest concentration (C–G).

**Figure 4:**
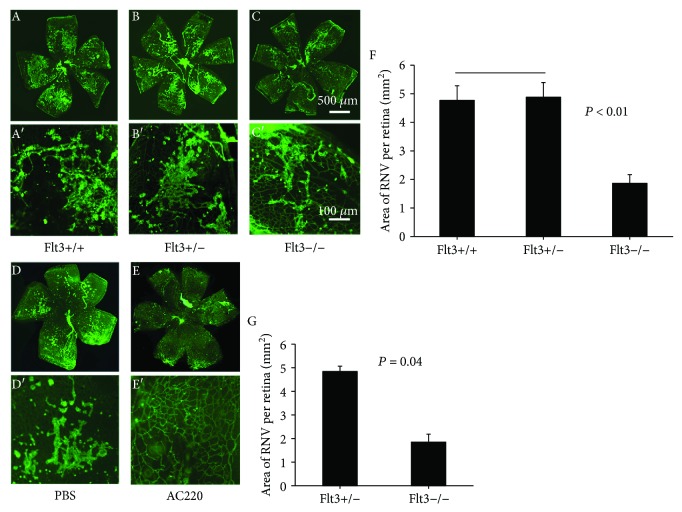
Ischemia-induced retinal NV decreases in Flt3-deficient animals. Flt3−/−, Flt3+/−, or Flt3+/+ (C57BL/6) mice were exposed to 75% oxygen from P7 to P12. At P18, retinas were dissected, washed, and incubated in presence of FITC-lectin at room temperature for 45 minutes. Retinal flat mounts were assessed under a fluorescence microscope. Retina samples from Flt3−/− animals (C, C′) showed reduced NV in comparison with those from the Flt3+/− (B, B′) or Flt3+/+ counterparts (A, A′). Quantitation of retinal NV areas confirmed a significant decrease of NV areas in the Flt3^−/−^ group (*n* = 12) compared with Flt3+/− (*n* = 12) or Flt3+/+ (*n* = 12) specimens (F). Immunofluorescence staining of retinal flat mounts of OIR animals after treatment with PBS and AC220. AC220 or PBS was intravitreally administered in at P12; retinas were obtained, incubated with FITC-lectin, and flat mounted at P18 (*n* = 12). Quantitation of retinal NV areas confirmed a significant decrease in the AC220 treatment group (*n* = 12) in comparison with the PBS group (G). Bars represented mean (±SEM); groups were compared by ANOVA with SNK for multiple comparisons.

Mice lacking Flt3 showed markedly reduced angiogenesis (Figures 3 and 4). Systemic neutralization of Flt3 in C57BL/6 mice starkly decreased CNV (Figure 3), as obtained with Flt3 gene deletion. Representative CNV lesions from an eye of wild-type animals (Figure 3(A)) and Flt3 transgenic mice (Figure 3(B)) showed profound differences in CNV. CNV areas were markedly decreased in Flt3−/− mice, as well as in animals administered 1.0 *μ*g/*μ*l or 10 *μ*g/*μ*l AC220; however, the animals treated with 0.1 *μ*g/*μ*l AC220 and controls showed similar values (Figure 3(E)). RNV areas were significantly decreased in Flt3−/− mice, as well as in animals administered 1.0 *μ*g/*μ*l AC220, compared with control values (Figure 4).

### 3.4. The Flt3 Inhibitor AC220 Significantly Reduces Subretinal NV and the Ratio of TRD in VEGF-Overexpressing Mice

**Figure 5:**
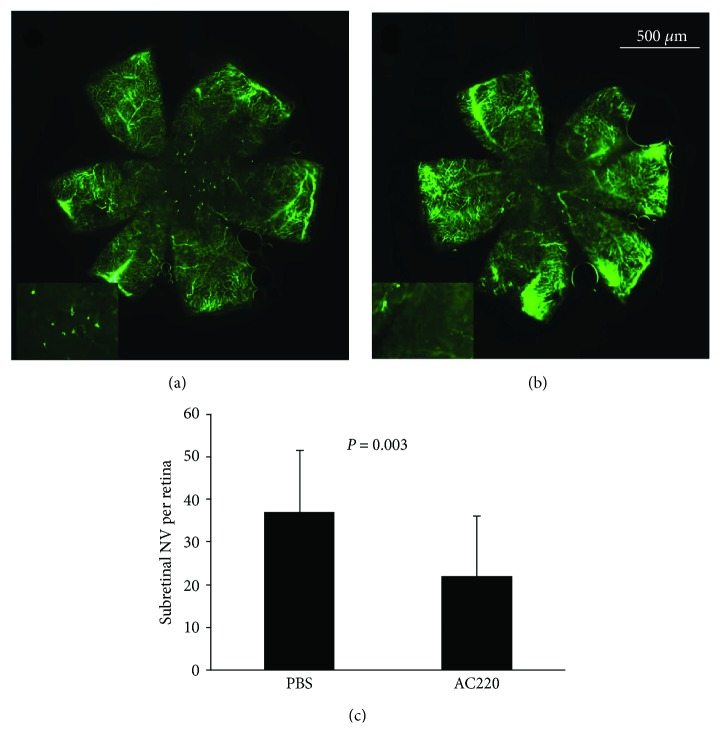
Inhibition of Flt3 reduces VEGF-induced subretinal NV. Immunofluorescence staining of retinal flat mounts from rho/VEGF animals after treatment with PBS and AC220. AC220 was intravitreally administered in one eye and PBS in the contralateral eye of rho/VEGF mice at P12, and retinas were obtained, stained, and flat mounted at P21 (*n* = 8). The total number and area of subretinal NV decreased significantly in AC220-treated eyes compared with those in the PBS group (a–c).

**Figure 6:**
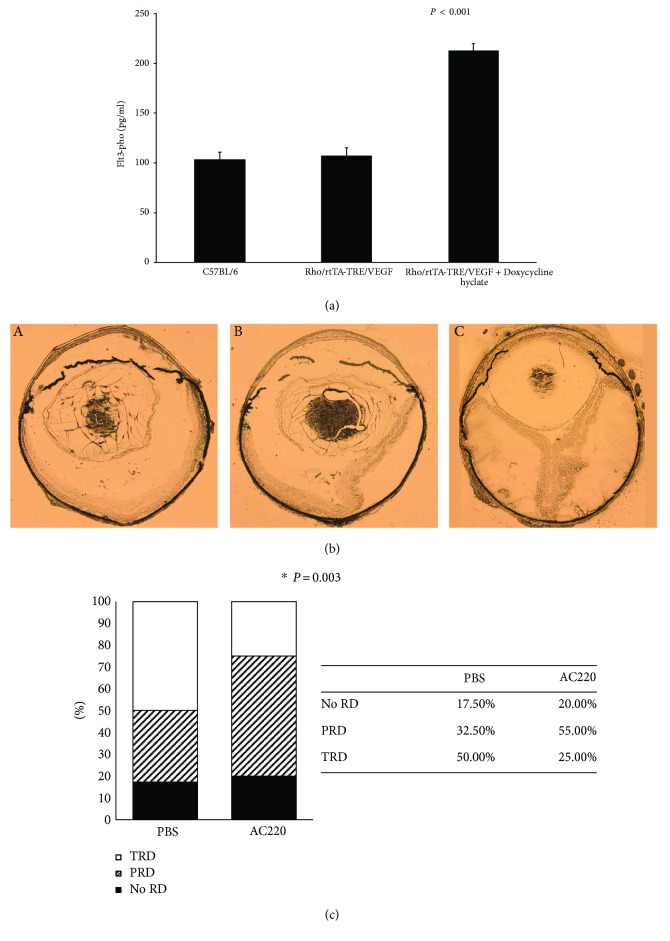
Effect of intraocular injection of AC220 in double-transgenic mice overexpressing VEGF in photoreceptors (rho/rtTA-TRE/VEGF mice). Four-week-old adult rho/rtTA-TRE/VEGF mice received an intravitreal injection of 1 *μ*g of AC220 (in 1 *μ*l PBS) in one eye and 1 *μ*l PBS in the contralateral eye. The animals then received doxycycline at 2 mg/ml in drinking water. (a) After five days, non-AC220-treated retinas were lysed, and phosphorylated Flt3 amounts were measured by the ELISA method. Phosphorylated Flt3 amounts in RD model retinas were higher than those in both wild-type and no-doxycycline-treated retinas. (b) Five days after initiation of doxycycline treatment, ocular sections showed no retinal detachment (no RD), partial retinal detachment (PRD), or total retinal detachment (TRD). (c) Intraocular injection of AC220 or PBS in doxycycline-treated rho/rtTA-TRE/VEGF mice. Retina samples were examined under an operating microscope in a blinded manner, to determine if there was TRD, PRD, or no RD. In AC220-treated mice, 25% of eyes had TRD, and 50% of eyes injected with PBS had TRD. The asterisk “∗” indicates that the incidence of RD in AC220-injected eyes was significantly reduced than that of the PBS-injected counterparts (*P* < 0.05 by chi-square). This suggests that intravitreal injection of 1 *μ*g AC220 significantly reduced TRD.

Retinal flat mounts assessed by fluorescence microscopy revealed that AC220 inhibited subretinal NV (Figure 5). Rho/rtTA-TRE/VEGF (Tet/opsin/VEGF) animals, compared with those with intraocular injection of 1 *μ*l of PBS and 1 *μ*g/*μ*l of AC220, respectively, showed significantly less TRDs (Figure 6), suggesting that VEGF-induced ocular NV was closely associated with Flt3.

### 3.5. Association of Flt3 with Macrophages

**Figure 7:**
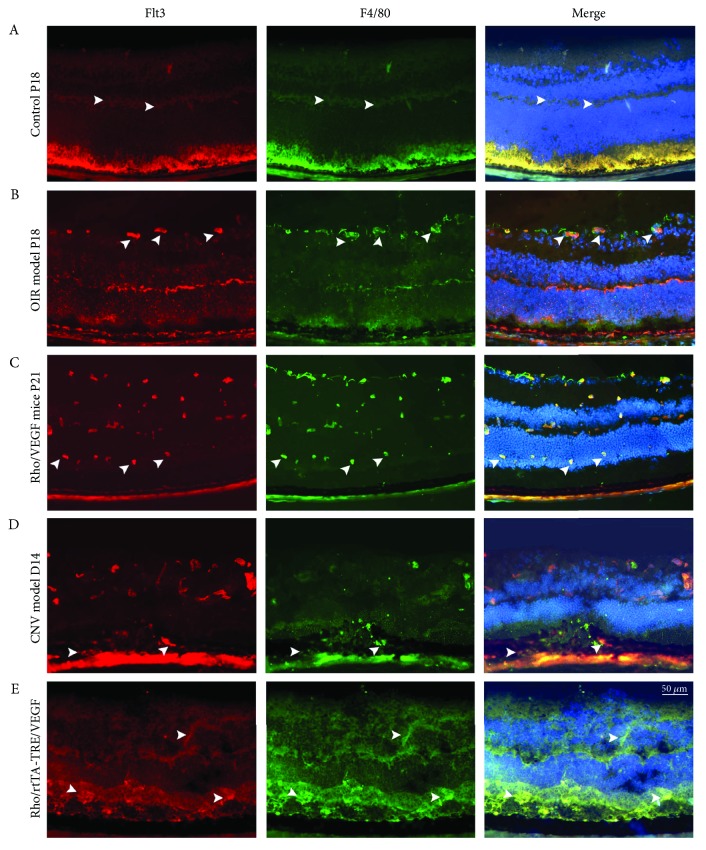
Immunofluorescence staining for Flt3 and F4/80 in normal, ischemic retina, rho/VEGF transgenic, CNV, and rho/rtTA-TRE/VEGF transgenic retinas. Retinal preparation was conducted for staining F4/80 macrophages and Flt3+ cells using the perfusion procedure described earlier. Eyes of OIR mouse model at P18, subretinal NV mouse model at P21, CNV and RD mouse models were enucleated and submitted to fixation and preparation for frozen sections. Immunofluorescence staining of Flt3 (red) showed high expression of Flt3 in OIR, subretinal NV, CNV, and RD model retinas (arrowheads), which colocalized with F4/80 staining. A nonischemic retinal sample from a P18 mouse displayed Flt3 expression areas, as revealed by Cy3-conjugated secondary antibodies (red), throughout the inner retina. F4/80 expression in the same section as in (A) with FITC-conjugated secondary antibodies displayed reduced areas. Ocular section from a P18 mouse after oxygen-induced ischemic retinopathy displayed areas (arrowheads) of increased expression in the whole inner retina colocalizing with F4/80-stained macrophages or closely localized around macrophages in and on the retina (B). Ocular specimens from P21 transgenic mice overexpressing VEGF in photoreceptors (rho/VEGF mice) showing Flt3 expression that colocalizes with F4/80 (C). An ocular section from a D14 CNV mouse model showing Flt3 staining, while F4/80 staining of the same section showed macrophage localization (D). Ocular sections from 5- to 8-week-old transgenic mice (rho/rtTA-TRE/VEGF) showing an area of Flt3 staining in the inner layer (red) and green F4/80 staining (E). Merging of Flt3 and F4/80 staining of all NV model retinas showed colocalization (yellow), indicating that macrophages may express Flt3.

Retinal sections were stained with F4/80 and Flt3 antibodies (for labeling of Mphi and Flt3 stained cells, resp.) and evaluated using fluorescence microscopy for visualization and colocalization of immunocompetent retinal cells. Retinas from P18 rodents with oxygen-induced ischemic retinopathy displayed areas of enhanced Flt3 expression throughout the inner retina compared with those of samples from normal P18 mice, with colocalization with F4/80 on the surface of the retina in new vessels (Figures 7(A, B)). Eyes from P21 transgenic animals overexpressing VEGF in photoreceptors (rho/VEGF mice) showed Flt3 expression in the inner and intermediate layers, with close association with F4/80 (Figure 7(C)). Ocular sections from D14 choroidal NV models or 5- to 8-week-old transgenic mice (rho/rtTA-TRE/VEGF) also showed areas of Flt3 staining, which were closely associated with F4/80 expression in the same layer (Figures 7(E) and 7(D)), indicating that Flt3 was strongly induced in retinal Mphi.

### 3.6. Flt3 Regulates M1 and M2 Cytokine Expression in CNV Mouse Model

**Figure 8:**
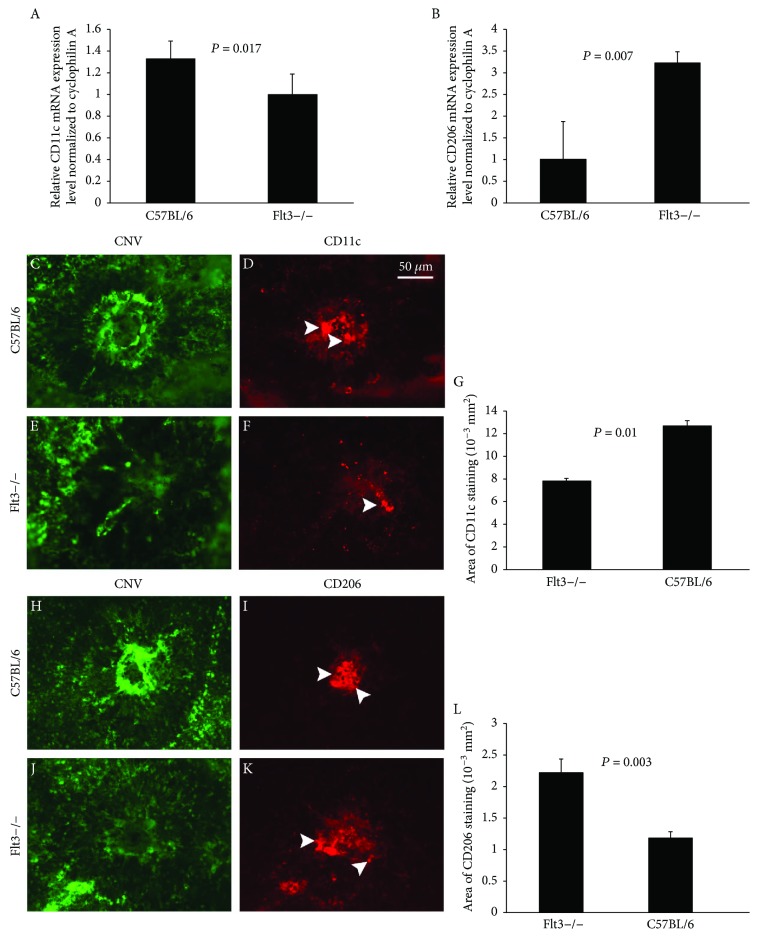
CD11c and CD206 levels assessed by quantitative PCR and immunofluorescence in retinas of CNV mouse model at D14. Quantification of CD11c and CD206 levels in mouse retinas are shown, respectively, in (A) and (B). Data are mean ± SE from three independent experiments (*n* = 8, *P* < 0.05). Flt3−/− and wild-type C57BL/6 mice were submitted to laser-induced CNV. Double labeling of NV (green) with CD11c (red) or CD206 (red) was performed on choroidal flat mounts of mice after laser treatment. Immunofluorescence signals of CD11c are shown in (D) and (F); CD206 are shown in (I) and (K). Mean CD11c(+) and CD206(+) cell areas per CNV lesion are presented. Flt3−/− mice exhibited significantly decreased areas of CD11c staining and increased areas of CD206 staining in comparison with controls (*P* < 0.05); similar results were obtained at the mRNA level by real-time RT-PCR.

Retinas of Flt3−/− and wild-type C57BL/6 mice at D14 with CNV were collected for the assessment of Mphi polarization-associated molecules. Spiller et al. demonstrated that M1 Mphi appear at early stages of wound healing (1–3 days), later followed by the M2 counterparts (4–7 days) [41]. Therefore, we examined the inflammation related effectors, including CD11c and CD206, most likely involved in M1 and M2 cytokines, respectively. The results suggested that Flt3−/− animals showed relatively reduced amounts of CD11c and elevated levels of CD206, with statistical significance comparable with that of wild-type controls (Figures 8(C, D)). This indicated a potential role for Flt3 inhibitors in Mphi polarization. Immunofluorescence staining of CD11c and CD206 in retina samples from CNV models at D14 also revealed that Flt3 promoted the expression of M1 cytokines (Figures 8(C–F)) and decreased that of M2 cytokines (Figures 8(H–K)). Quantification of CD11c+ staining areas in CNV lesions at 14 days confirmed a substantial decrease in the amounts of these cells in the absence of Flt3 (Figure 8(G)). CD206+ staining areas were significantly increased in the Flt3−/− group (Figure 8(L)).

### 3.7. Flt3 Deficiency Shifts Macrophage Polarization towards the M2 Phenotype during RNV

**Figure 9:**
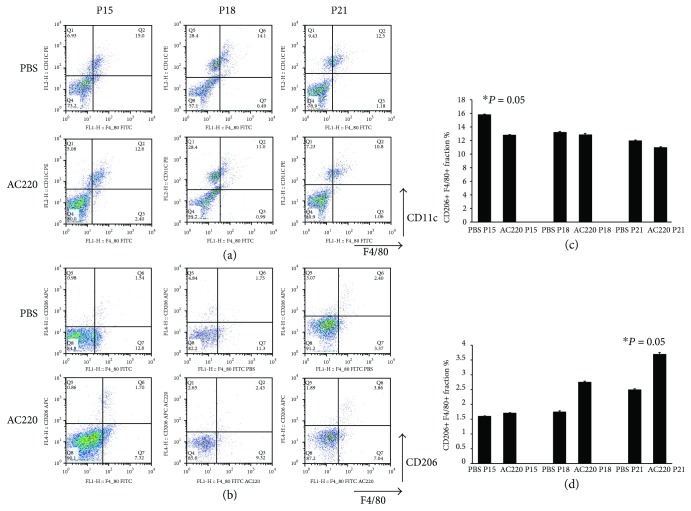
Flow cytometry analysis of PBS and AC220-treated OIR mouse model. PBS and AC220 treatments were administered to OIR model mice. At P15, P18, and P21, retina samples were digested with papain, and cells were sorted by CD11b expression before incubation with anti-F4/80, CD11c, and CD206 antibodies. M1 and M2 macrophages were F4/80+/CD11c+ and F4/80+/CD206+, respectively. Representative flow cytograms are shown (A and B). Cycle distribution patterns of F4/80+/CD11c+ and F4/80+/CD206+ cells (C and D). The asterisk “∗” indicates a significant change of F4/80+/CD11c+ fraction or F4/80+/CD206+ fraction in AC220 treatment mouse retina specimens compared with PBS control samples. Bars represented mean ± SE from 3 independent experiments (*n* = 4–6 mice/group, *p* < 0.05).

Retina samples from AC220- or PBS-treated eyes were assessed by flow cytometry (P15, P18, and P21). There were reduced amounts of M1 Mphi in AC220-treated animals compared with PBS controls (P15 showed more striking differences); however, more M2 Mphi were found in AC220-treated animals compared with PBS controls, with P21 showing more pronounced effects. M1/M2 ratios indicated a shift in Mphi polarization towards M2 in the AC220 group compared with the PBS group in RNV (Figure 9).

## 4. Discussion

The area of neovascular complexes penetrating the subretinal space reflects the degree of angiogenesis in CNV. NV pathogenesis has been evaluated by established mouse models, which serve as a tool for preclinical trials assessing therapies for AMD [27, 42]. In this study, inflammation was further characterized by evaluating CNV induction in Flt3-deficient mice. Our working hypothesis was that reduced inflammation and CNV would be found in Flt3-deficient mice. As demonstrated by our findings, mice lacking Flt3 showed a significant decrease in new vessel growth in both transgenic mice and Flt3 inhibitor AC220 injection group. CNV results from damage to Bruch's membrane and RPE defects. In contrast, RNV results from retinal hypoxia, which is found in various ocular diseases, including diabetic retinopathy and retinopathy of prematurity [43]. Interestingly, injection of the Flt3 inhibitor AC220 resulted in substantially decreased NV areas in our RNV models.

Inflammatory cells, specifically Mphi, produce multiple angiogenic factors and constitute important components in malignancies, heart disease, and ocular disease. However, Mphi functions are rather complex as they induce wound healing [44, 45] while controlling angiogenesis during development [31]. Tsutsumi et al. demonstrated that ocular-infiltrating Mphi were essential for CNV generation, with the influx of Mphi possessing direct angiogenic ability [46]. Caicedo et al. proposed that recruitment of blood-derived Mphi seemed to be more associated with CNV than resident microglia [47].

Flt3 can influence the functions of Mphi. Flt3 ligand exerts potent stimulatory effects on precursors of the monocyte/Mphi lineage [48]. In one study, Flt3 ligand caused whitening of the bone marrow with significant reduction in the number of erythroblastic island Mphi and erythroblasts [49]. Furthermore, Flt3 ligand might be considered a specific marker for IFN*γ*-differentiated Mphi. Polarizing cytokines such as IFN*γ* might contribute to the high levels of Flt3 ligand found in rheumatoid arthritis synovium by shifting the Mphi polarization into a M1 phenotype [50]. In our study, we demonstrate for the first time that Flt3 deficiency results in Mphi switching towards the M2 phenotype in addition to decreased NV. This shows that targeted therapy with the Flt3 inhibitor AC220 is able to reduce local Flt3 function, in turn influencing tissue-specific M2 activity and the controlling NV formation. Mphi are found in two polarization phenotypes, M1 and M2 subsets, which promote and inhibit inflammation, respectively [51, 52]. Brichard et al. demonstrated that M2 cells regulate inflammation and promote tissue repair [53], in turn increasing trophic rescue with heightened clearance of apoptotic cellular debris and promoting tolerance in lieu of autoimmunity [54, 55]. A report by Cao et al. also revealed that an increased number of M2 cells in normal aging eyes compared to M1 Mphi facilitates tissue repair and NV [56].

Transgenic mice with the rhodopsin promoter controlling human VEGF165 levels in photoreceptors (rho/VEGF mice) produce new vessels from the retina's deep capillary bed, starting at P10, which grow into the subretinal space [25, 40]. VEGF production is sustained, and therefore, the new vessels continue to grow and enlarge, forming large nets in the subretinal space similar to the exudative type of AMD [57]. In a retinal detachment model, the Tet/on system was employed to produce double-transgenic mice characterized by doxycycline-inducible expression of human VEGF165 in photoreceptors (rho/rtTA-TRE/VEGF or Tet/opsin/VEGF mice) [26]. A dose of 2 mg/ml doxycycline in drinking water highly enhanced VEGF levels in photoreceptors (similar to but higher than those in rho/VEGF mice), causing total exudative retinal detachment in 80–90% of mice within 5 days. Cryosections of retinas were obtained for further assessment from the experimental group and controls [58, 59]. Experiments involving the Flt3 inhibitor AC220, in this study, provided evidence of an association of Flt3 activation with VEGF-induced RNV. Notably, the inhibition of Flt3 induction significantly decreased VEGF-induced RNV in both rho/VEGF and rho/rtTA-TRE/VEGF animals. Furthermore, Flt3 inhibition with AC220 reduced the retinal detachment rate in rho/rtTA-TRE/VEGF mice. These findings support the notion that inhibition of Flt3 by AC220 affects retinal new vessel formation in the retina and detachment induced by VEGF in these models. Markovic et al. showed that the Flt3-blocking antibody D43 decreased Flt3 tyrosine phosphorylation and reduced Flt3-induced VEGF secretion, indicating that Flt3 signaling is highly involved in VEGF regulation [60].

Further studies are needed to determine the potential mechanisms by which Mphi are polarized in Flt3 deficiency, how Flt3 modulates retinal NV during VEGF overexpression in transgenic mice, and whether Flt3 upregulation can increase NV areas in RNV models. Our study highlights Flt3 as a novel molecular target for the inhibition of neovascularization in murine models of retinopathy. Further evaluation of the antiangiogenic role of Flt3 inhibition in human eyes is needed.

## 5. Conclusion

In this study, we demonstrated that Flt3 inhibition improved ocular NV by driving Mphi switching towards the M2 phenotype. Our findings revealed Flt3 effects on NV via Mphi polarization modulation, supporting its potential use for the treatment of NV in ocular disease.
